# Vaccination as a preventative measure contributing to immune fitness

**DOI:** 10.1038/s41541-021-00354-z

**Published:** 2021-07-27

**Authors:** Béatrice Laupèze, Giuseppe Del Giudice, Mark T. Doherty, Robbert Van der Most

**Affiliations:** 1 GSK, Wavre, Belgium; 2 GSK, Siena, Italy; 3 GSK, Rixensart, Belgium

**Keywords:** Immunology, Vaccines

## Abstract

The primary goal of vaccination is the prevention of pathogen-specific infection. The indirect consequences may include maintenance of homeostasis through prevention of infection-induced complications; trained immunity that re-programs innate cells to respond more efficiently to later, unrelated threats; slowing or reversing immune senescence by altering the epigenetic clock, and leveraging the pool of memory B and T cells to improve responses to new infections. Vaccines may exploit the plasticity of the immune system to drive longer-term immune responses that promote health at a broader level than just the prevention of single, specific infections. In this perspective, we discuss the concept of “immune fitness” and how to potentially build a resilient immune system that could contribute to better health. We argue that vaccines may contribute positively to immune fitness in ways that are only beginning to be understood, and that life-course vaccination is a fundamental tool for achieving healthy aging.

## A new role for vaccines

The global population is aging. As a result of effective infectious disease prevention in infancy and early childhood in well-resourced countries, the greatest proportion of the burden of vaccine-preventable diseases is now in older adults: for example, the number of adults who die from a vaccine-preventable disease in the United States is now 350-times higher than the number of such deaths in children^1^. As populations age, “Healthy aging”, defined by the World Health Organization (WHO) as “the process of developing and maintaining the functional ability that enables well-being in older age”^2^ is becoming critical to social and economic sustainability in the twenty-first century. Accordingly, achieving ‘Healthy Aging’ is becoming a major public health focus^2^.

Infant and early childhood vaccination programs in place since the 1940s brought about wide-ranging benefits to societies by preventing death and disability due to previously common childhood infections, with substantial longer-term positive economic impacts^3^. In the twenty-first century, the role of vaccination is evolving, broadening from a primary focus on prevention of untimely death, to that of improving overall health and well-being throughout life^4^. This approach, often called life-course vaccination, aims to allow individuals to profit from disease prevention and the indirect health benefits associated with vaccination throughout all stages of life^5^.

Globally, the vast majority of people have received at least some vaccines during infancy, childhood, adolescence, and/or as adults. Vaccines contribute to health by preventing otherwise potentially disabling or fatal diseases. But do they contribute in other ways? Can the other, indirect effects of vaccines on the immune system contribute to long-term health? Although aging is accompanied by an immune decline (immunosenescence), the immune system maintains some degree of plasticity and diversity in response to external challenges, even in old age. In this article, we discuss the concept of “immune fitness” and how to potentially build a resilient immune system for better health. Two important features of the immune system potentially capable of modulating immune resilience in older age are innate trained immunity as a result of epigenetic re-programing^6,7^, and expansion of the repertoire of T cell and B cell responses which becomes limited with increasing age. We argue that vaccines may contribute positively to immune fitness, loosely defined as the ability to adapt to external challenges by mounting and regulating an appropriate immune response, in ways that are only beginning to be understood, and that life-course vaccination could be a fundamental tool for achieving healthy aging.

## The nature and determinants of healthy aging

**Fig. 1:**
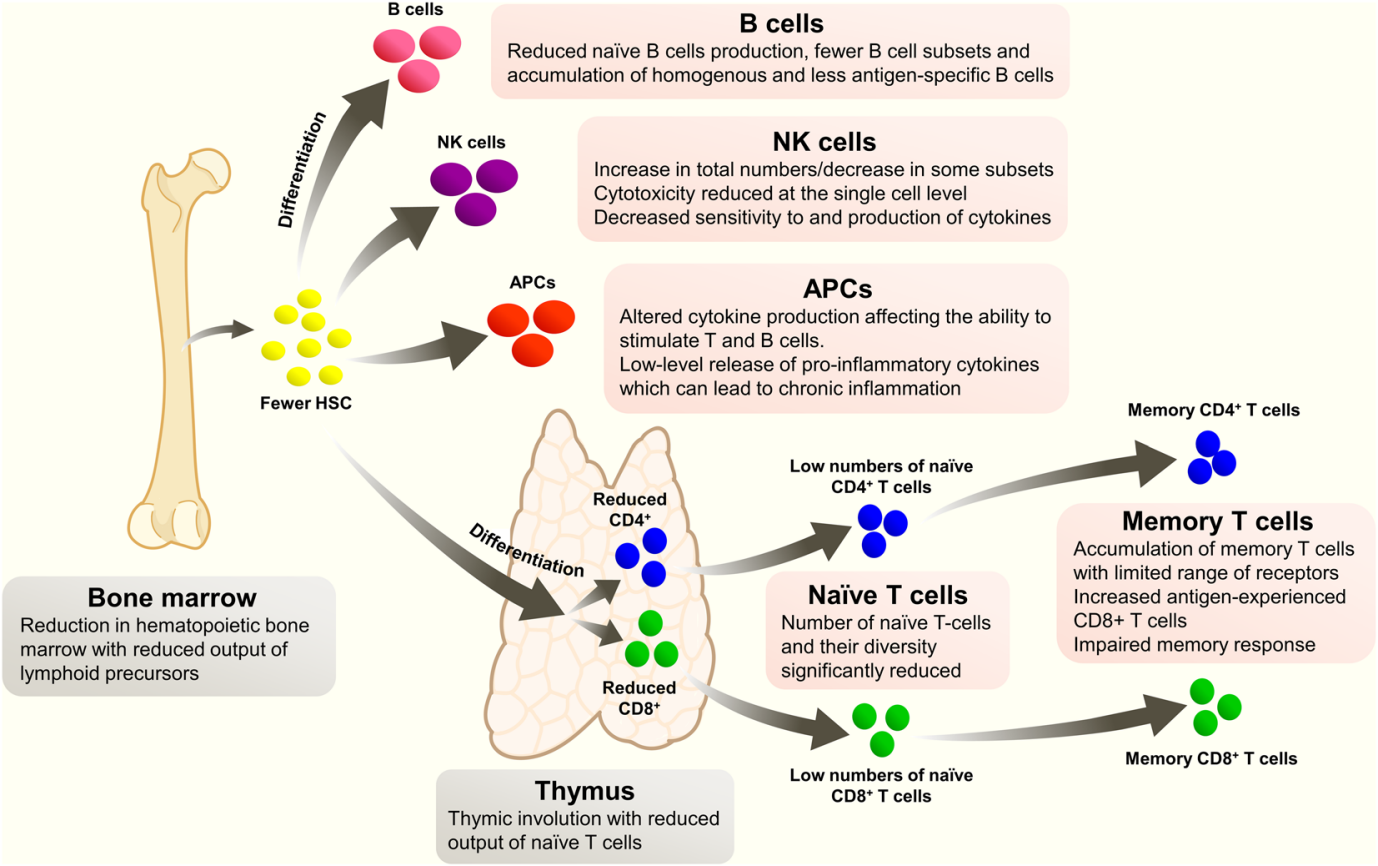
Changes in the immune system with age. Summary of key changes in the immune system with age.

Immunosenescence refers to the gradual global decline of multiple aspects of the immune system that accompanies the normal aging process (Fig. 1)^8^. Structural components of the immune system such as the bone marrow and thymus involute and reduce in volume, and their output of naive immune cells markedly decreases with age. T cell and B cell responses are reduced, with fewer available precursors and a higher proportion of homogenous memory cells. Epigenetic dysregulation in B and T cells, characterized by DNA methylation and changes to histone expression and acetylation, is thought to contribute to a pro-inflammatory environment (‘inflammaging’), with an increased tendency to broadly reactive, non-specific stimulation that promotes the development of chronic inflammation, autoimmune diseases, and cancer (Box 1)^8–11^. Epigenetic re-programming during aging may also reduce the ability to effectively respond to immune challenges such as infection or vaccination^10^.

The pace and extent of immunosenescence is not fixed and the immune system may retain a high degree of plasticity during older age. In mice, specific deficits in cellular glycolysis and mitochondrial one-carbon metabolism were identified in aged T cells^12^. Significantly, however, the addition of the products of one-carbon metabolism improved T-cell activation and functioning ex vivo, suggesting that some of the cellular alterations that accompany immune senescence can be reversed or prevented.

While chronological age is strongly associated with immune health, it is not necessarily the most important factor. An individual’s biological age (bioage) is a more accurate indicator of remaining healthy life span and active life expectancy^8,13,14^. Bioage is a function of numerous intrinsic and extrinsic influences such as genetic make-up, prior and existing diseases, environmental exposures (toxins, medicines, smoking, alcohol, etc.), diet, physical activity, and other lifestyle behaviors and exposures^8^. Epigenetic biomarkers of aging (‘epigenetic clocks’) based on DNA methylation levels have been constructed by Horvath, Hannum, and Levine^15^. Levine’s DNAm PhenoAge epigenetic biomarker correlates with all-cause mortality, morbidity, external factors such as diet, body mass index, exercise, and education level, and includes an immune signature that is consistent with the inflammaging hypothesis^16^. However, as yet, standardized and validated methods to reliably quantify bioage using biomarkers or functional body measures are not available^14,17,18^. Nevertheless, estimates of bioage have been shown to be predictive of responses to vaccination, survival, and cognitive functioning in older age^14,17–19^. While both genetic and environmental factors shape bioage, the influence exerted by environmental factors affects the rate of change in bioage, and the effect of those influences increase with age^18,20^. This suggests that bioage is amenable to external manipulation at the epigenetic level, with potentially positive or negative effects.

The immune status of any individual is the sum of their genetic make-up and all of the external influences they have experienced, i.e., their immunobiography^11^. The immune system is highly plastic, able to recognize and respond to continual internal and external immune challenges^11^. The immunobiography can therefore be thought of as the sum of all the responses to the type, dose, and sequence of antigenic exposures that occur during an individual’s lifetime. Each infection, vaccination, and environmental exposure contributes to the education of the innate and adaptive immune systems which underlie the responses to all subsequent exposures. This vast variability is reflected in the highly heterologous immunity capacity observed in adults and is evidence of the constantly evolving immune system^21^.

Immune phenotypes vary markedly between individuals of the same age, including monozygotic twins who are genetically identical. Twin studies have provided vital clues as to the influence of environment on immune phenotype. Although commencing life with very similar epigenetic profiles, over time, the levels of DNA methylation and histone acetylation diverge markedly in twins, probably due to different environmental influences and epigenetic ‘drift’, resulting in increasingly different cellular phenotypes over time^22^. In monozygotic twins, variations in immune responses to influenza vaccination, and variations in populations of specific cells, serum levels of interleukins, and other serum proteins, are largely a result of non-heritable influences and become more marked with increasing age^20^.

The list of environmental influences that shape the immunobiography is practically limitless^23^. Aside from age and sex, known strong influences are chronic infections caused by cytomegalovirus (CMV), human immunodeficiency virus (HIV), or tuberculosis, and the microbiome—itself known to be intricately linked to immune system homeostasis^24^. The microbiome continuously evolves in response to external factors, such as diet, the ingestion of drugs and pathogens, and other environmental exposures: for example, proximity to pets, livestock, toxins, and physical activity^16,25,26^. Dysbiosis (an imbalanced microbiome) is a pathogenic event associated with hyperimmune responses in the gut, increased gut permeability and perturbations of the systemic immune response^25^. The development or exacerbation of numerous inflammatory diseases such as inflammatory bowel disease, multiple sclerosis, rheumatoid arthritis, diabetes, atopic dermatitis, asthma, as well as obesity, metabolic syndrome, and colorectal cancer have all been associated with dysbiosis and loss of immune integrity of the intestinal mucosa^26^.

The immune system is thus programmed by environmental exposures that have long-term implications for health^23,27^. It therefore follows that guided exposures could potentially modulate the immune system to maximize beneficial health outcomes, and that it should be possible to take advantage of the adaptability of the immune system to improve life-long immune-mediated health^27^.

Box 1: Epigenetics and aging Epigenetics refers to inherited or induced changes that involve alteration of gene expression rather than changes to the genomic sequence. The epigenome controls access of transcription factors to promoter and silencer regions within DNA, and thus regulates transcription. Histones manage the structure of chromatin, and modifications to histones through acetylation or methylation at Lys and Arg sites, or phosphorylation, alter the chromatin structure and modifies transcription. Certain epigenetic marks (patterns of chemical groups added to DNA and histones) are relatively stable throughout life^107^. Other regions are highly susceptible to changes in the environment, and can alter rapidly in response to environmental modifications caused by internal and external factors such as diet, drugs, smoke, stress, hormones, and circadian rhythm^107^. Changes to the epigenome directly impact cellular phenotype and can thus directly influence states of health or disease.

The major epigenetic changes that occur with aging are a progressive decrease in DNA methylation with an expression of some DNA sequences that are normally silenced, and a general loss and redistribution of heterochromatin^107,108^. In parallel, localized hypermethylation can silence normally active gene promoters^10^. Histones may lose repressive marks while gaining activating marks. The accumulation of epigenetic changes in immune cells is thought to contribute to immune dysregulation through loss of control of gene expression and abnormal activation of transcription^10^. In T cells, epigenetic changes associated with aging resemble the changes associated with activation, which could explain the reduced capacity of T cells to respond to new threats in older persons^10^.

## Immune fitness

Immune fitness can be defined as a state in which an individual’s immune system is resilient, having an intrinsic capacity to adapt to external challenges by developing and regulating an appropriate immune response^28^. Resilience is the capacity of the immune system to return to homeostasis after an external challenge. In other words, immune fitness is the ability to mount the minimum appropriate immune responses required to deal efficiently with the source of immune stimulation, ensuring the individual stays in good health. In much the same way that exercise contributes to a general state of physical fitness, immune fitness can be seen as a general state that does not guarantee health, but reduces the risk of ill-health.

**Fig. 2:**
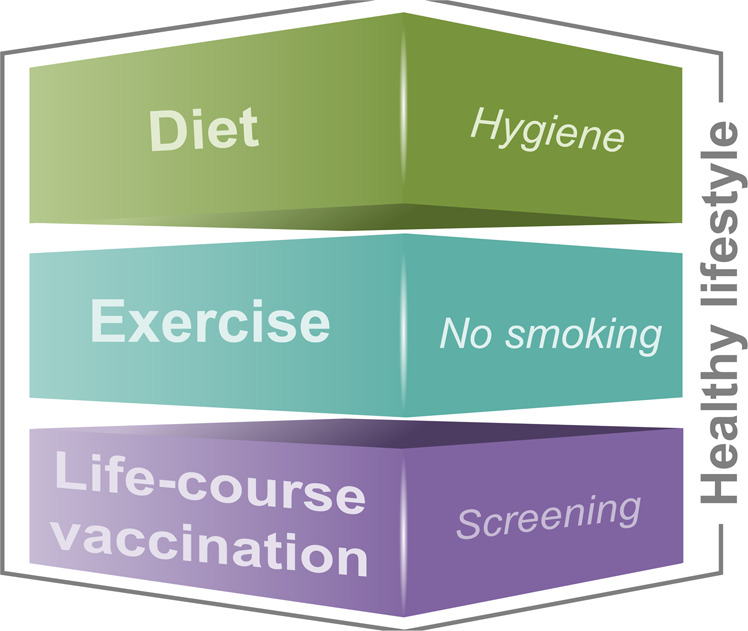
Healthy lifestyle pyramid adapted from Philip et al.^5^ Life-course immunization as a key component of a healthy lifestyle pyramid.

Building a “fit” immune system relies to a certain extent on genetic predisposition, but external factors that positively influence and train the immune system towards long-term health appear to be more important^20^. Education of the immune system begins before birth through maternal diet and intra-uterine exposure to antibiotics, pathogens, or toxins. Later on, the method of delivery, breastfeeding, exposure to pets and livestock, inheritance of microbiota from household members, and exposures to antibiotics, drugs, and toxins, all affect the developing immune system and influence responses to future antigenic exposures, including vaccination^29–32^. Important known environmental influences on the immune system in adulthood are diet, exercise, the presence of chronic infection, and exposures to common pollutants such as smoking. Three of these four are to a great extent under the control of the individual, are relatively easily amenable to change, and are included in the healthy lifestyle pyramid proposed by Philip et al.^5^, who also included life-course immunization as a key component for building immune fitness (Fig. 2).

A healthy diet is central to maintaining a healthy microbiome, which in turn is critical to immune homeostasis, preventing colonization and invasion by pathogens, promoting tolerance to colonizing bacteria, and maintaining the integrity of the gut mucosal barrier^33^.

Exercise can have substantial positive effects on the adaptive and innate immune systems^34^. In older people, exercise increases T cell proliferation, increases the number and function of neutrophils and NK cells, improves the function of monocytes/macrophages, and appears to correct the pro-inflammatory state associated with immune senescence through reduced secretion of pro-inflammatory cytokines^34,35^. Exercise immediately before vaccination appears to improve the immune response, and higher seroprotection rates to influenza vaccines were observed in older persons who undertook a 10-months cardiovascular training program^36,37^.

During chronic infections such as hepatitis B, hepatitis C, and HIV, an antigen is continually presented to the immune system, leading to continuous immune stimulation of virus-specific CD8^+^ T cells that can become functionally exhausted^38^. Immune exhaustion is characterized by long-term proliferation of T cells in response to the continued presence of antigen, with loss of effector and memory functions, resistance to apoptosis, and accelerated immune senescence related to telomere attrition^38^. One of the most common chronic infections is CMV, a ubiquitous herpes virus with a latent “smoldering” stage that can only be controlled by constant immune surveillance^39^. Controlled CMV infection (maintaining a latent state) appears to confer some immune benefits including improved responses to influenza vaccines and a reduced risk of some cancers^40^. However, the persistent low-grade immune activation that accompanies CMV re-activation from latency causes clonal expansion of CMV-specific CD8^+^ T memory cells with impaired replicative capacity, reduced receptor diversity, and reduced ability to respond to new threats^39^. These changes are similar to those observed in immunosenescence and it has been suggested that CMV acts to hasten biological aging in infected individuals^41^. Moderate exercise appears to improve immune control of CMV, although it is not yet known if existing changes to the immune system induced by CMV can be reversed by exercise^40^.

Vaccines impact the immune system both directly and indirectly. Their most studied direct effects are the induction of antigen-specific immune responses able to protect against the diseases targeted. It is also known that vaccines can have indirect effects on other illnesses, although quantifying these effects and consciously manipulating them to the advantage of the individual is not yet common practice^42^.

The “positive side-effects” of vaccination have long been recognized. Numerous empirical studies from the 1800s reported that smallpox vaccination caused improvements in rashes and chronic infections, and made individuals less susceptible to measles, scarlet fever, whooping cough, and syphilis^43^. Originally termed “para-immunity”, this phenomenon was explored in pox viruses by Mayr et al. in Germany who identified increases in some cytokines and mediators of specific and downstream immune responses after vaccination with highly attenuated animal pox viruses^43^. It was proposed that vaccines that induced para-immunity could be used to optimize the immune response, either through activation, regulation, or suppression, for prophylactic or therapeutic purposes^43^.

## Trained immunity

**Fig. 3:**
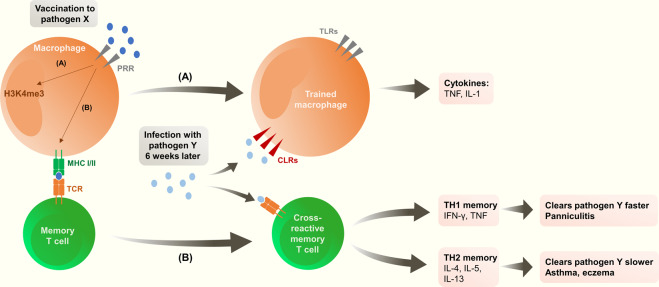
Possible immunological mechanisms explaining downstream effects of vaccination (adapted from Benn 2013). After vaccination for pathogen X two possible pathways may explain downstream effects: **A** Epigenetic re-programming of monocytes/macrophages leading to a more rapid activation after exposure to pathogen Y, to ensure rapid clearance of this pathogen. **B** T cell-mediated cross-reactivity: memory CD4 and CD8 T cells are generated that are cross-reactive with pathogen Y. PRR pattern recognition receptors, MHC major histocompatibility complex, TLR Toll-like receptors, CLR C-type lectin receptors, TCR T cell receptor.

The innate immune system was previously believed incapable of developing or maintaining long-term immune memory. We now know that innate cells (e.g., monocytes, macrophages, natural killer [NK] cells, etc.) can be re-programmed by specific immune stimuli, which can cause them to respond differently to later exposures (Fig. 3)^6,44^.

Unlike memory induced in the adaptive immune response, trained immunity is not based on clonal expansion but on a relatively stable functional re-programming of innate cells that primes them to be more or less reactive to certain stimuli. Exposure to immunogenic stimuli can cause energy metabolism in innate cells to switch from oxidative phosphorylation to aerobic glycolysis, with other changes in cholesterol synthesis, glutamine metabolism, and potentially other as yet unidentified pathways^7^. Metabolic change can result in epigenetic re-programming through modifications to histones, DNA methylation, and reconfiguration of chromatin, and changes to the expression of pattern recognition receptors (PRRs) on the cell surface^7,45^. Histones associated with promoters of genes responsible for the production of pro-inflammatory cytokines and reactive oxidative species are modified by acetylation, which leads to increased transcription, whereas methylation typically inhibits transcription^44^. Thus, the nature and magnitude of the immune stimulus determine whether the trained immune response will be one of immune tolerance or immune paralysis to reduce tissue damage, or of hyper-inflammation to improve responses to threats including infections and cancers^6^. As an example, Bacillus Calmette–Guérin (BCG) and β-glucan can train the innate immune response to augment the response to unrelated stimuli, whereas, depending on dose, repeated exposure to lipopolysaccharide may induce immune tolerance with reduced capacity to respond to future simulation^45^. Compared to adaptive immune responses, trained immunity is relatively short-lived and is thought to last months rather than years^46^, but nevertheless lasts far longer than classical innate immunity for which the duration is typically a matter of hours. Trained immunity appears to be maintained by direct re-programming of hematopoietic stem and progenitor cells in the bone marrow that allows transmission of their phenotype down their lineage^46^.

Thus, it is reasonable to hypothesize that modulation of trained immunity might be exploited as a therapeutic tool: for example, to either reverse immunotolerant states that can lead to diseases such as sepsis or malignancy, or to increase pro-inflammatory responses on exposure to infectious agents^47^.

Trained immunity is one mechanism that has been suggested to explain the impact of some infections and vaccines beyond the disease targeted. The impacts of BCG vaccination or measles vaccination on overall health have been most widely studied in this context.

BCG vaccination has positive heterologous effects on mortality, cognitive development, and cancer incidence. All-cause mortality among children under 5 years of age was reduced by 30% after BCG vaccination in clinical studies, and by 53% in observational studies, an effect which was well beyond that which could be explained by the prevention of tuberculosis alone^48^. Recently, a 60-years follow-up of participants of a placebo-controlled BCG vaccine trial that commenced in 1935 in the United States reported that the incidence of lung cancer in BCG recipients was 2.5-fold lower than placebo recipients, and was not associated with prior tuberculosis infection^49^.

BCG vaccination at birth has heterologous influences on the magnitude of responses to subsequent routine vaccines administered in infancy. One study in Australia showed higher antibody levels in infants who received BCG at birth to subsequently administered tetanus toxoid and to pneumococcal and *Haemophilus influenzae* type b polysaccharides in conjugate vaccines, but lower responses to hepatitis B^50^. By contrast, an earlier study in the Gambia found that administration of BCG at birth or at the time of primary vaccination increased antibody and cell-mediated responses to hepatitis B vaccine, but had limited or no impact on responses to tetanus or diphtheria toxoids^51^. These apparently discordant results could be a result of underlying differences in ethnicity, primary vaccination schedule (acellular pertussis vaccines in Australia versus whole-cell pertussis vaccines in the Gambia) or other factors, and highlight the challenges in untangling the numerous potential influencers of immune responses.

Two meta-analyses, conducted at the request of WHO, concluded that BCG vaccination is very likely to have beneficial downstream effects. Nevertheless, the quality of some of the studies was questioned, and it was stressed that more research is required to conclusively show a link^48^.

On the basis of early observations that Covid-19 morbidity and mortality appear to be lower in countries with a current or recent policy for universal BCG vaccination^52,53^, studies are currently underway to test the hypothesis that trained immunity induced by BCG could provide some protection against Covid-19.

The mechanism of BCG-induced trained immunity appears to be via modification of H3K4me3 promoters of genes that encode IL-6, TLR4, and TNFɑ in NK cells and macrophages (Box 2)^45^. BCG increases the expression of specific PRRs on monocytes, and the expression of pro-inflammatory cytokine responses to pathogenic bacteria, fungi, and viruses is enhanced in BCG-vaccinated individuals, persisting for at least 3 months after vaccination^54,55^. Moreover, among healthy individuals who received a yellow fever vaccine as a model of a viral human infection, those who had received the BCG vaccine 1 month earlier had significantly lower viremia and improved anti-viral responses compared with volunteers who received placebo vaccination^56^.

BCG was also the first approved biological therapy and continues to be used as a successful treatment for bladder cancer via immune-modulating effects that result in the direct cytotoxic killing of cancer cells^57^.

Measles vaccines also appear to be particularly potent in training the innate immune response (Table 1). A meta-analysis found that all-cause mortality in children after receiving a measles-containing vaccine was significantly lower (49% reduction) in 18 observational studies (although only smaller, non-significant effects have been observed in clinical trials which are typically not powered with non-disease-specific effects in mind)^48^. In contrast, acute measles infection has long been associated with an increase in all-cause mortality in the years after infection, probably as a result of measles-induced immunosuppression through lymphocyte repression and depletion that can last for several years after wild-type infection^58^. The opposing effects of vaccination versus natural infection on the immune system likely reflect differences in the type or magnitude of the immune response induced including marked cell death among lymphocytes during measles infection that is not observed after vaccination^59^, possibly because vaccine and wild-type measles strains are recognized by different PRRs; TLR2 for wild-type measles versus TLR3 for vaccine strains^60^. The clear survival benefit associated with measles vaccination thus appears to be due to the combined effect of preventing acute measles illness and the subsequent prolonged period of immunosuppression and susceptibility to infection, and the indirect effect of trained immunity that enhances, rather than depresses, responses to other pathogens.

**Table 1:** Downstream effects of vaccination.

	Observation	Potential mechanism
Vaccinia virus^60,61^	• Reduced all-cause mortality • Protection against melanoma and non-Hodgkin lymphoma • Anecdotal associations with improvements in rash, syphilis, chronic diseases • Reduced nasopharyngeal carriage of *Streptococcus pneumoniae* and *Haemophilus influenzae*	• Upregulation of monocyte/macrophage markers with increased expression of pro-inflammatory cytokines and of genes involved in cell metabolism and trained immunity
BCG^48,59,60^	Reduced all-cause mortality • Reduced sepsis, respiratory infections, respiratory syncytial virus infection, and fever in low birth weight infants • Enhanced antibody responses to other vaccines • Effective treatment against bladder cancer • Enhanced resistance to bacterial, fungal, and viral infections in mice • Prevention of development of type 1 diabetes • Increased susceptibility to *Salmonella typhi* or *Eberthella typhosa*	• Lymphocyte-mediated effects including CD4^+^ and CD8^+^ cells modifications leading to enhanced Th1/Th17 responses during non-related infections • Increased activity of NK cells • NOD2-mediated epigenetic re-programing of H3K4me3 with enhanced transcriptional activity in pro-inflammatory promoter regions
Measles-containing vaccine^48,49^	• Reduced all-cause mortality • Reduced nasopharyngeal carriage of *Streptococcus pneumoniae* and *Haemophilus influenzae*	• Transient suppression of lymphoproliferative responses but increased non-specific cytokine production (IL-6, TNFɑ, and IFNƴ) suggesting increased innate immune responses
Yellow fever vaccine^59,60^	• Reduced all-cause mortality	• Activation of mTOR with evidence of changes to metabolism and upregulation of histone methylation.
Rotavirus vaccine^61,105,106^	• Reduced risk of developing autoimmune diseases including type 1 diabetes and celiac disease in the period after vaccination • Reduced risk of seizure	• Not known
Formalin-activated respiratory syncytial virus vaccine^62,106^	• Enhanced respiratory disease on re-exposure	• Excessive Th2-biased response

These downstream benefits of vaccination are limited neither to measles vaccination nor to vaccination in childhood. A study of a cohort of adults in Guinea-Bissau vaccinated against smallpox between 16 and 18 years of age found a 40% lower risk of all-cause mortality compared to individuals who did not have a smallpox scar, and who were therefore assumed to be unvaccinated. In this case, the effect cannot be explained by the prevention of smallpox, since the disease had been eradicated long before the study was conducted^61^.

Not all downstream effects are positive. For example, the immune response induced by a formalin-inactivated respiratory syncytial virus vaccine tested in children during the 1960s led to much more severe respiratory disease on re-exposure to RSV^62^. Much less is known about the downstream effects of other vaccines and the findings are not always clear-cut, in part because of the difficulty of controlling for bias in observational studies^48,63^ and this remains an area of ongoing study.

Adjuvants are stimulants used in vaccines to enhance the immune response. Their role was historically to increase the magnitude of the antibody response to vaccines containing antigen with limited inherent immunogenicity^64^. Traditionally, adjuvant development was empirical. Now, however, the mechanisms of action of adjuvants are being unraveled, and the development of new adjuvants and Adjuvant Systems (combinations of immune stimulants) has opened the door to a wider role for adjuvants in directing and enhancing antibody and cellular immune responses. Adjuvant Systems such as AS01 used in the Recombinant Zoster Vaccine (RZV: *Shingrix*, GSK), and AS04 used in the recombinant hepatitis B vaccine (*Fendrix*, GSK), and the recombinant human papillomavirus vaccine (*Cervarix*, GSK), use PRR signaling through TLR4 and exert their effects by optimization of the innate immune system^65,66^. MF59 and AS03 are oil-in-water emulsions containing squalene (AS03 also contains vitamin E) that are used in seasonal and/or pandemic influenza vaccines. MF59-adjuvanted seasonal influenza vaccine is licensed for adults over the age of 60 years. MF59 and AS03 activate immune pathways and dendritic cells through TLR-independent mechanisms^67–69^.

The AS01-containing RZV indicated for the prevention of herpes zoster in adults aged 50 years and older is the first vaccine to induce age-independent protection, and shows that the age-related decline in immunity in very old adults can be overcome^70^. A hepatitis B vaccine containing CpG, a toll-like receptor (TLR9) agonist, was subsequently licensed in the US (*Heplisav-B*, Dynavax). This adjuvanted vaccine also showed improved immunogenicity in older persons, with 91.6% of 60–70 year-olds achieving seroprotection compared to 72.6% vaccinated with an alum-adjuvanted hepatitis B vaccine^71^. Whether AS01, CpG or the emulsion adjuvants induce trained immunity requires further investigation. An intriguing hint is provided by the observation that innate immune responses, such as those linked to interferon-gamma, are stronger after a second dose of an adjuvanted vaccine as compared to the first dose^72^. Trained immunity has been proposed as one potential explanation^72^. Interestingly, adjuvanted seasonal influenza vaccines provided greater efficacy than unadjuvanted vaccines in preventing hospitalization due to pneumonia/influenza^73^ or all-cause mortality and pneumonia^74^. An ongoing Phase I clinical study is investigating (i) the effects of RZV vaccination on trained immunity and (ii) whether this could result in less disease associated with influenza, pneumonia, and Covid-19 (NCT04523246).

The role of adjuvants in shaping immune memory has been demonstrated for influenza vaccines. In the face of antigenic diversity, the process of generating B cell adaptability is driven by cross-reactive CD4^+^ memory cells, such as antigen-specific T follicular helper cells derived from previous infections or vaccinations. B cell adaptability to heterologous strains is substantially enhanced when primary vaccination against the initial strain is with an adjuvanted influenza vaccine^75^. AS03 and MF59-adjuvanted influenza vaccines stimulate CD4^+^ T cells and naive B cells targeting a broad range of epitopes with the production of antibodies with diverse binding sites and increase avidity compared to the non-adjuvanted vaccine, with increased adaptability of memory B cells for greater specificity to the new strains (epitope spreading)^76–78^. In practice, these findings strongly suggest that altering the microenvironment in which antigen recognition takes place has profound consequences for subsequent immune responses, and that the ‘correct’ vaccination can induce CD4^+^ T cell responses that prepare the immune system for a more effective response, even to antigens from heterologous disease strains.

Consistent with these findings, it has been argued that as a result of age-related declines in naive T cell numbers, function, and diversity of repertoire, cross-reactive memory T cells serve as increasingly important mediators of protection against new infections in older adults^79^. Less stringent requirements for activation of memory T cells implies that immune responses mediated by cells with lower avidity recruited from the memory pool might be delayed, weak, and potentially highly restricted due to the effects of clonal cell expansions in response to chronic infections (e.g. CMV) and the confines of the individual immunobiography^79^. The diversity of the B cell repertoire is similarly depleted over time, with clonal expansion of antigen-experienced cells^80^. Some vaccines, such as the live attenuated zoster vaccine, have been shown to expand the T cell repertoire in older adults^81^, and one of the functions of adjuvants in vaccines is to increase the breadth of the immune response^76,77^. Therefore, strategies such as choosing adjuvants capable of enhancing T memory cell adaptability could be of particular benefit to older adults who have a limited pool of naive T cells, and in whom pre-existing memory B cells could be leveraged to generate high-affinity antibodies with the help of pre-existing T cells.

Infections are frequently followed by complications that may not be recognized as related, but which are due to immune modifications induced by the infection, or which are the result of the additional stress of infection on body systems that may already be compromised. For vaccine-preventable diseases, prevention of these downstream effects contribute significantly to the value of vaccination, and are increasingly recognized as important for cost-effectiveness analyses and economic modeling of the impacts of vaccination^82^.

For example, individuals with herpes zoster are at significantly higher risk of stroke, particularly in the first 4 weeks post-infection, and of cardiac events for more than a year after infection^83–85^. Respiratory diseases and deaths due to circulatory disease all increase during influenza seasons^86–88^, and influenza vaccination, particularly of older adults, impacts positively on hospitalization rates for respiratory and cardiovascular diseases, and reduces all-cause mortality^89,90^.

Childhood vaccination induces positive health benefits that manifest as significantly improved cognitive abilities in teenagers compared with unvaccinated controls, probably due to fewer infections during childhood^91^. Thus, vaccination can be considered an intervention that acts to maintain homeostasis by preventing a primary disease (the targeted infection) and secondary disease (other illnesses arising as a result of the primary infection). In the absence of vaccination, infections by vaccine-preventable diseases can have major, long-term flow-on effects, impacting the cognitive development of children, reducing their life-long productivity, and increasing the risk of a host of other diseases, mainly respiratory, cardiovascular, and cerebrovascular, with the attendant morbidity, mortality and associated healthcare and economic costs.

Box 2: Epigenetic modifications induced by BCG BCG induces trained immunity via the PRR nucleotide-binding oligomerization domain-2 (NOD2). NOD2-mediated signaling of the protein kinase B–mammalian target of rapamycin–hypoxia-inducible factor-1α (AKT/mTOR/HIF-1ɑ) pathway induces modifications to metabolic pathways including upregulation of aerobic glycolysis and epigenetic rewiring^6^. Modification of histone 3 occurs with the addition of three methyl groups (trimethylation) to lysine 4 (H3K4me3), which is involved in the regulation of gene expression^109^. H3K4me3 regulates promoters of genes that encode IL-6, TLR4, and TNFɑ in NK cells and macrophages^45^. BCG re-programming in the bone marrow results in modification of transcription in hematopoietic stem cells with enhanced production of myeloid cells and production of educated monocytes improved protective capabilities^110^. Increased expression of specific PRRs on monocytes, and the expression of pro-inflammatory cytokine responses to pathogenic bacteria, fungi, and viruses are enhanced in BCG-vaccinated individuals for up to 1 year after vaccination^54,55^.

## Life-course vaccination for immune fitness and healthy aging

**Fig. 4:**
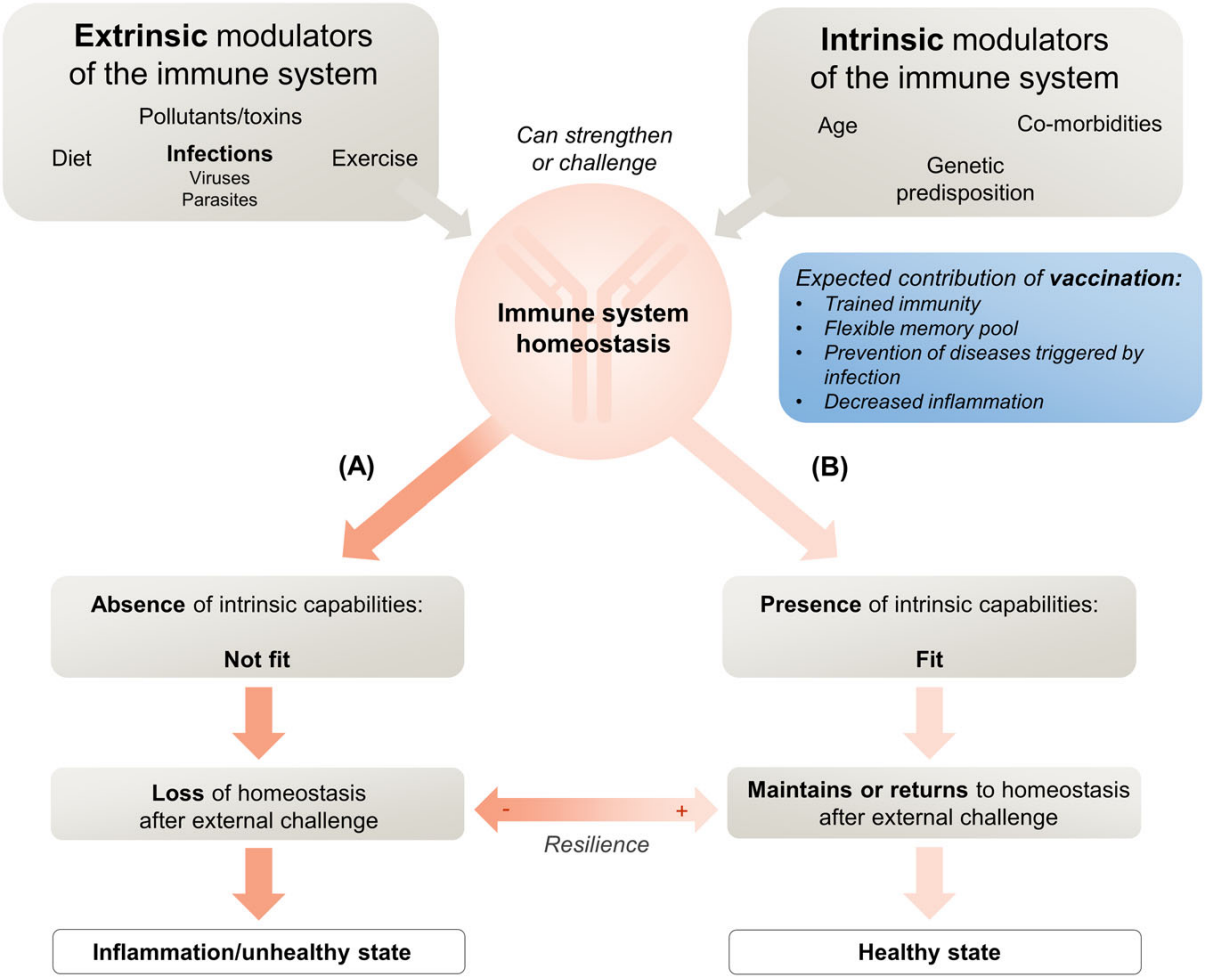
Intrinsic and extrinsic modulators of immune fitness. Intrinsic and extrinsic modulators may either challenge or strengthen the immune system. External challenges can result in either **A** the loss in immune system homeostasis (unhealthy state) in the absence of intrinsic capabilities or **B** the maintenance of homeostasis in the presence of intrinsic capabilities (healthy state). Vaccination may potentially have a positive influence through trained immunity, by creating a flexible memory pool, by direct and indirect prevention of diseases triggered by infections and associated inflammation.

It is clear that the external influences of diet, exercise, combined with good hygiene, and the avoidance of toxins such as smoking have far-reaching effects on health including immune health. We now understand that vaccination is an immune event of similar significance, that can educate and modulate the immune system, modifying responses to subsequent, and possibly unrelated, exposures^21^. Vaccines can make many-faceted contributions to healthy aging that can act positively and/or negatively according to age, sex, immunobiography, and characteristics of the vaccine itself, including the presence of adjuvant and vaccine sequence. Infant vaccination is an early modulator of immunobiography and the administration of vaccines to train innate immunity at key points throughout life has the potential to prevent immune senescence and to positively impact healthy aging. The positive impact of vaccines on the epigenetic clock is expected to be indirect, mainly mediated by the prevention of chronic viral infections which have been described to accelerate biological age measured by the epigenetic clock^41,92–94^. As yet, however, the planned use of specific vaccinations to leverage the immune system toward optimal overall health remains theoretical (Fig. 4). Likewise, the prevention of infectious diseases throughout life, either by direct effects or by the downstream effects of vaccination, appears to contribute to preventing the negative consequences of inflammation associated with those diseases, but has not yet been applied intentionally for this purpose. While the impact of low-grade, chronic inflammation associated with older age on vaccine responses has been described^11^, little is known whether vaccination can directly impact inflammation by prevention or modulation of maladaptive immune responses. Preliminary evidence suggests that drugs acting directly on mechanisms involved in the process of aging may improve fitness, as evidenced in two studies by increased responses to influenza vaccine, upregulation of antiviral gene expression, and lower infection rates among older adults treated with an mTOR inhibitor^95,96^. Inhibition of sestrins (stress sensing proteins) leading to broadly enhanced T cell activity is another intervention showing early promise^97^.

## Conclusion

Current vaccination programs primarily focus on childhood vaccination but the evidence discussed here indicates that they need to evolve so that the benefits from the pathogen-specific and downstream effects of vaccines are available to all individuals throughout their life. Rather than the traditional lens of targeted age-based vaccination requirements, life-course vaccination views vaccination as a life-long, continuous activity, akin to exercise, that aims to build and maintain better overall health^98^. Vaccines can potentially positively impact immune fitness via innate and adaptive responses that improve and maintain the resilience of the immune system, either by epigenetic re-programing of innate cells or by leveraging pre-existing memory established by previous immunizations. Vaccines maintain immune homeostasis by preventing the targeted disease and the associated morbidity and death triggered by infection with long-term impacts on health.

The current Covid-19 pandemic has exposed the extreme vulnerability of older persons to severe infectious diseases. Understanding the specific elements of the immune system that increase the vulnerability of the aged compared to younger persons is critical to identify effective treatments or vaccines against Covid-19, and is equally applicable to the prevention and treatment of other infectious diseases^99^. The intriguing observation that prior vaccination against influenza or pneumococcus was associated with a statistically significantly lower probability of a positive test for SARS-CoV-2 in older adults lends credence to the notion that vaccination can confer wider benefits than pathogen-specific immunity^100^. We are in the midst of a revolution in immunology, made possible by the analysis of immune responses at the single-cell level using advanced technologies such as T cell receptor sequence analysis (assay for transposase-accessible chromatin with sequencing, or ATAC-seq), and epigenetic profiling of histone modifications using cytometry by Time-Of-Flight (EpiTOF)^101^. Growing understanding of the intricate mechanisms that underlie human health and disease is opening the door to interventions that could target immune fitness.

In conclusion, there is a growing need for a paradigm shift in how vaccination is viewed and promoted. Outside of their traditional role, vaccines could contribute positively to immune fitness in ways that are only beginning to be understood, although how that contribution might be made needs more study. Confirmation of the downstream effects of vaccines would strongly support the role of vaccination in health promotion and the need to add vaccination to the toolbox for healthy living; together with a healthy diet, exercise, and smoking cessation^102–104^.

## Data Availability

No datasets were generated or analysed during the current study.
